# Correlates of cervical cancer screening uptake among female under graduate students of Aksum University, College of Health Sciences, Tigray, Ethiopia

**DOI:** 10.1186/s13104-019-4570-z

**Published:** 2019-08-19

**Authors:** Dawit Gebregziabher, Eskedar Berhanie, Tsiyon Birhanu, Kidanemariam Tesfamariam

**Affiliations:** 1 Nursing School, College of Health Sciences and Comprehensive Specialized Hospital, Axum University, Tigray, Ethiopia; 2 Department of Medical Laboratory, College of Health Sciences and Comprehensive Specialized Hospital, Axum University, Tigray, Ethiopia

**Keywords:** Cervical cancer screening, Correlates, Uptake

## Abstract

**Objectives:**

Cervical cancer is among the leading cancer related causes of morbidity and mortality of women in the world. Ethiopia is among the highest risk countries with age adjusted incidence of cervical cancer 35.9 per 100,000 women or 7619 new cases and 6081 deaths of cervical cancer each year. The aim of this study was to examine correlates of cervical cancer screening uptake among female under graduate students of Aksum University, College of Health Sciences. Data was collected using self-administered structured questionnaire. Variables that were statistically significantly associated with the outcome in bivariate analyses were considered in a logistic multivariate regression analysis.

**Result:**

Only 17.2% of students were screened for cervical cancer in their lifetime. Both in bivariate and multivariate analysis, sexual experience (AOR = 38.85; 95% CI [8.907, 169.51]), marital status (AOR = 3.481; 95% CI [1.167, 10.380]), Ppace of birth (AOR = 3.359; 95% CI [1.559, 7.235]), and student’s year of study (AOR = 0.005; 95% CI [0.001, 0.031]) were the only correlates of cervical cancer screening uptake among female students. Therefore, the overall cervical cancer screening uptake was low and further study should be done other correlates.

## Introduction

Cervical cancer (CC) is a malignant tumor of the cervix affecting the outermost squamous or inner glandular cells [1]. It is the fourth most common cancer in women, worldwide [2]. Cervical cancer is highly linked to preventable sexually acquired infection with certain types of HPV (16 and 18) [3]. HPV infection has been shown as the primary agent of cervical cancer and it can be diagnosed through routine screening based on clinical history and/or physical findings and confirmed by cervical punch or cone biopsy [4].

Cervical cancer is common among women worldwide. Most cases that occur in developing countries are related to the availability of screening and to human papillomavirus vaccination programs [5]. Screening can detect precursors and early-stage disease for both types of cervical cancer: squamous cell carcinoma and adenocarcinoma. Treatment of precursors and early-stage disease can prevent the development of invasive cervical cancer and reduce cervical cancer mortality [6]. Cervical cancer screening began with the development of the Papanicolaou (Pap) test. In countries that adopted Pap test screening, the incidence and mortality of cervical cancer have decreased. In addition to the Pap test, screening methods now include tests for high-risk strains of HPV, which are central to the pathogenesis of cervical cancer. Infection with high-risk strains of HPV and persistence of HPV infection are the most important correlates of progression to cervical cancer [7].

An estimated 70,722 new cervical cancer cases occur annually in sub-Saharan Africa, which was the highest in the world with age standardized incidence rate of 31 per 100,000 women and varies across the regions with 42.7 in east Africa and 29.3 in western [8]. Ethiopia is among the highest risk countries for cervical cancer and populations of 29.43 million women aged 15 and above are at greater risk to develop cervical cancer [9].

Cervical cancer screening is the process of detection of precancerous cervical lesions in healthy women before the lesions develop into cancer [3, 10]. These precancerous lesions can be treated easily [11]. Cervical cancer screening tests are available globally and the screen and treat is one simplified approach that has been developed and accepted by WHO. It uses visual inspection with acetic acid (VIA) and immediate cryotherapy for secondary prevention of cervical cancer [10, 12]. This approach has been adopted and is now being used in countries with limited resources such as Ethiopia.

According to WHO, cervical cancer screening is aimed at all females in the target age group, followed by treatment of detected precancerous lesions so that the majority of cervical cancers are prevented. WHO further recommend that at least one cervical cancer screening be performed on any female who is 30 to 49 years old. However, this can be extended to females younger than 30 years provided there is evidence of a high [11].

The female University students of were chosen to participate in the study because they are mostly in the age group (18 to 30 years), which poses a greater risk of HPV infection. In addition, female students in the health field are responsible for primarily informing the individual on health issues and they also play a key role in raising awareness on the risks of cervical cancer [13]. Female students should especially be sensitive about female cancers such as cervical cancer, so that they can play a professional role in protecting and promoting community health as healthcare personnel. This study was aimed to examine correlates of cervical cancer screening uptake among female under graduate students of Aksum University, and to assist program planners and health educators to target and tailor prevention programs.

## Main text

### Materials and methods

An institutional based cross-sectional study was conducted among female under graduate students of Aksum University, College of Health Sciences from February to June 2018. Aksum University College of Health Science was established in May 2016. It was started giving service in 2016 and the hospital has 201 beds for admitted patients but there are 177 beds are currently provided service for patients. It is expected to serve 3.5 million people from 3 different zones of the Tigray region.

The source populations for this study were all female undergraduate students of Aksum University. All sampled female under graduate students were also studied population for this study. A multistage cluster sampling technique with help of random sampling was used to select a total of 344 female health sciences students of the Aksum University. All under graduate female students who were off campus during the study period were excluded from the study.

The student’s cervical cancer screening practice for premalignant cervical lesion was measured their self-report history of screening in the past 3 years. Students were asked (“Have you ever screened for cancer of the cervix?”) Response options were binary: 1 = Yes, 2 = No. Further, they were also asked about the procedure used for screening, Response options according to the screening modalities examined in this study were 1 = VIA, 2 = Pap smear, 3 = Biopsy 4 = I do not know?

Data was collected using standardized self-administered structured questionnaire. It was entered using Epi info version 7, cleaned and analyzed using Statistical package for social sciences version 22.0 software. Descriptive statistics was used to characterize the sample. The binary logistic regression model was employed to examine the statistical association between the outcome variable and selected independent variables. All variables with a P value < 0.05 were included in the multivariable analysis using default variable selection method. Ethical clearance was secured from institutional review board (IRB) of Aksum University, College of Health Science. Written consent was obtained from each participant. Information was recorded anonymously and confidentiality were assured throughout the study period.

### Result

#### Socio-demographic characteristics of respondents of Aksum University

In this study, a total of 344 students completed the questionnaire making the response rate of 100%. The mean ( ± SD) age of students was 23.67 ± 2.83 years’ ranging from 19 to 30 years. Two hundred sixty-one (75.9%) of students came from urban and the rest 261 (75.9%) were from rural setting. Regarding marital status, 28 (8.1%) were married. Only 205 (59.6%) had sexual experience, of those 132 (38.4%) have had Multiple sexual partner. Regarding year of education, 55 (16.0%) were first year students, 55 (16.0%) second year, and 166 (48.3%) were fourth year students and above. Two hundred forty-six (71.2%) were Orthodox, 50 (14.5%) protestants (Table 1).

**Table 1:** Socio-demographic characteristic of female under graduate students of Aksum University, College of Health Science, Tigray, Ethiopia (N = 344)

Variables	Category	Frequency (%)
Age of respondent	19–24	249 (72.4)
	25–30	83 (24.1)
Marital status	Single	254 (73.8)
	Married	28 (8.1)
	In relationship	62 (18.0)
Sexual experience	Yes	205 (59.6)
	No	139 (40.4)
Age at fist sex	15–20	150 (43.6)
	> 21	56 (16.3)
Number of sexual partners	None	139 (40.4)
	Single	73 (21.2)
	Multiple	132 (38.4)
Place of birth	Urban	261 (75.9)
	Rural	83 (24.1)
Level of education	Year I	55 (16.0)
	Year II	55 (16.0)
	Year III	68 (19.8)
	Year IV and above	166 (48.3)
Religion	Orthodox	246 (71.2)
	Catholic	21 (6.1)
	Muslim	27 (7.8)
	Protestant	50 (14.5)

#### Knowledge about prevention, treatment and screening modalities of cervical cancer

More than one-third 140 (40.7%) students knew that cervical cancer could be prevented by avoiding multiple sexual partners, 56 (16.3%) reported that HPV Vaccination prevent cancer of the cervix. Less than one-fourth 71 (20.6) of respondents do not know whether it is treatable or not, Of those, who responded that cervical cancer is treatable 98 (28.5%) students said that cervical cancer can be treated by specific drug given by hospital.

Students were asked about the cost of treatment, 130 (37.8%) answered it is free of charge, and 117 (34%) did not know the cost of cervical cancer treatment. Regarding how frequent one should be screened for cervical cancer, 123 (35.8%) students answered once a year and 105 (30.5%) every 3 years. One hundred eighty-eight 188 (54.7%) answered that women of above 25 years of age should be screened, while 91 (26.5%) said that prostitutes should be screened (Table 2).

**Table 2:** Knowledge about prevention, treatment and screening modalities of cervical cancer of female under graduate students of Aksum University, College of Health Science, Aksum, Tigray, Ethiopia (N = 344)

Knowledge variable	Category	Frequency (%)
Prevention methods	Avoiding multiple sexual partners prevent cervical	140 (40.7)
	Avoiding early sexual intercourse	88 (25.6)
	Quitting smoking prevent cervical cancer	24 (7.0)
	Vaccination HPV prevent cervical cancer	56 (16.3)
	Don’t know	36 (10.5)
Know cancer of cervix can be treated	Yes	273 (79.4)
	No	71 (20.6)
Treatment type	Herbal remedies	44 (12.8)
	Specific drug given by hospital	98 (28.5)
	Surgery	126 (36.6)
	Radiotherapy	62 (18)
	Don’t know	14 (4.1)
How expensive do you think is treatment cervical cancer	Free of charge	130 (37.8)
	Reasonable price	25 (7.3)
	Moderately expensive	23 (6.7)
	Very expensive	49 (14.2)
	Do not know	117 (34)
Frequency of screening	Once a year	123 (35.8)
	Every three year	105 (30.5)
	Do not know	116 (33.7)
Who should be screened	Women of > 25 years	188 (54.7)
	Prostitutes	91 (26.5)
	Elderly women	65 (18.9)
Procedures used in cervical cancer screening	VIA	182 (52.8)
	Pap smear	44 (13.7)
	Biopsy	67 (19.5)
	Do not know	48 (14)

#### Cervical cancer screening uptake

Two hundred eighty-five (82.8%) of participants were not screened for cervical cancer. Only 17.2% were screened in their lifetime. The most frequent reason for not screening was lack of decision to be screened 72 (20.9%), 50 (14.5%) said that it may be pain full, and 31 (9.0%) said they feel shy.

#### Correlates of cervical cancer screening uptake

As can be noted from (Table 3), three of the seven variables which showed significant association with cervical cancer screening uptake in bivariate analysis could not persist as significant in the multivariable analysis.The results of multivariant analysis showed that after adjusting for number of sexual partners and age at first sex, only marital status, sexual experience, level of education and place of residency had showed overall significant association with cervical cancer screening uptake at 5% level of significance (Table 3).

**Table 3:** Bivariate and multivariable logistic regression analysis result of determinants of cervical cancer screening among female under graduate students of Aksum University, College of Health Science, Aksum, Tigray, Ethiopia (N = 344)

Variables	Practice		COR (95% CI)	AOR (95% CI)
	Yes	No		
Age of respondent				
19–24	49 (83.1.0%)	200 (70.2%)	1	1
25–30	10 (16.9%)	85 (29.8%)	2.968 [1.707, 5.159]*	0.989 (0.337, 2.902)
Sexual experience				
Yes	45 (76.3%)	160 (56.1%)	1	1
No	14 (23.7%)	125 (43.9%)	4.189 [2.537, 6.917]*	38.855 [8.907, 169.51]**
Marital status				
Single	46 (78.0%)	208 (73.0%)		1
Married	6 (10.2%)	22 (7.7%)	2.669 (1.046,1.808)*	3.481 [1.167, 10.380]**
In relationship	7 (11.8%)	55 (19.3%)	1.927 (1.046,3.550)*	1.365 (0.345, 5.396)
Place of birth				
Urban	43 (72.9%)	218 (76.5%)	3.672 [2.194, 6.144]*	3.359 (1.559, 7.235)**
Rural	16 (27.1%)	67 (23.5%)	1	1
Level of education				
Year I	12 (20.3%)	43 (15.1%)	1	1
Year II	23 (39.0%)	32 (11.2%)	0.041 [0.013, 0.132]*	0.005 [0.001, 0.031]**
Year III	10 (16.9%)	58 (20.4%)	0.669 [0.321, 1.392]	1.117 [0.400, 3.122]
Year IV (above)	14 (23.7%)	152 (53.3%)	2.393 [1.210, 4.732]*	1.463 [0.763, 5.274]

*P value < 0.05 in bivarite analysis

**P value < 0.05 in multivariable analysis

As a result, having previous sexual experience increases the odds of cervical cancer screening uptake by 38 times as compared to those who had never had sexual experience. (AOR = 38.86, 95% CI 8.907, 169.51). In addition, second year female students were 99.5% less likely to have been screened compared to fresh female undergraduate students (AOR = 0.005, 95% CI 0.001, 0.031).

Married participants were 3.4 times more likely to be screened compared with Single participants (AOR = 3.481, 95% CI 1.167, 10.380). female students who had been born in urban area were also 3.3 times more likely to be screened compared to participants who were born in rural area (AOR = 3.359, 95% CI 1.559, 7.235).

### Discussion

This study was aimed to examine correlates of cervical cancer screening uptake among female under graduate students in Aksum University. Of the 344 female students, only 59 (17.2%) had cervical screening in their lifetime. This is higher than the study findings of MizanTepi (Ethiopia) 14.83% ([14], 12.0% in Ghana [15], 9.0% from (Benin) [16], 0% in India and 1.0% Tunisia [17]. But it is lower than study conducted in 50.9% (Barbados) [17], in Uganda (19%) [18].The main reasons mentioned for not screened were lack of information, being healthy, shyness, did not decided to be screened, fear of vaginal examinations and pain.

In this study female students who have previous sexual experience had thirty-eight times higher odds of cervical cancer screening as compared to those without sexual experience (AOR = 38.86, 95% CI 8.907, 169.51). This finding is in line with a study conducted in 25 Low- and Middle-Income Countries (AOR = 3.62, 95% CI 2.98, 4.39). But in contrary to what was found in Adama University (Ethiopia) [17, 19]. The possible reason for these inconsistencies is difference in sample size and study population.

In addition, those Married participants were 3.4 times more likely to be screened compared with Single participants (AOR = 3.481, 95% CI 1.167, 10.380). The findings of the present study are consistent with those of earlier study from 25 Low, Middle Income and Emerging Economy Countries (AOR = 3.20, 95% CI 2.50, 4.09) [17].

This study also showed that female student’s year of study was also significantly associated with the screening practice of respondents. It was observed that second year female students were 99.5% times less likely to have been screened (AOR = 0.005, 95% CI 0.001, 0.031). This is in agreement with a study conducted in Hawassa University (AOR = 0.06, 95% CI 0.02, 0.17) and Adama University [19, 20]. This may be due to the influence of student’s level of exposure to different medical courses which in turn affect their screening practice.

Students place of birth were also significantly with their screening practice. This study has revealed that, being born in urban area were also three times more likely to be screened than their rural counterpart (AOR = 3.359, 95% CI 1.559, 7.235). This is in line with a study conducted in Adama University [19]. This could possibly be due to differences in the population’s exposure to technology, culture, and the participant’s residence area.

### Conclusion

In conclusion, this study revealed that female student’s cervical cancer screening uptake was low and sexual experience, place of birth, students class level and marital status were the only correlates of cervical cancer screening uptake. Further study with large sample size should be done to examine other correlates.

## Limitation of the study

Since our population interest were young female university students, and this has implications for the generalizability of the findings to less educated or older women. In addition, the cross-sectional nature of the study means causal inferences cannot be made from the results reported. Yet another notable limitation were very wide confidence intervals for some of the covariates and lack of adjustment for lifestyle factors.

## Data Availability

All the necessary data and materials of this study are readily available in the supplementary material section of the journal of BMC in Research Note.

## Abbreviations

AOR: adjusted odds ratios
CC: cervical cancer
CI: confidence interval
COR: crude odds ratios
HPV: human papillomavirus
IQ: interquartile
Pap smear: papanicolaou smear
SD: standard deviation
WHO: World Health Organization
